# Anthroponymy evolution of Javanese diaspora names in Malaysia (social onomastics study)

**DOI:** 10.3389/fsoc.2023.1292848

**Published:** 2023-12-11

**Authors:** Sahid Teguh Widodo, Suyatno Suyatno, Bahtiar Mohamad, Shafinar Ismail

**Affiliations:** ^1^Faculty of Cultural Sciences, Universitas Sebelas Maret, Surakarta, Central Java, Indonesia; ^2^Othman Yeop Abdullah Graduate School of Business (OYAGSB), Universiti Utara Malaysia, Kuala Lumpur, Malaysia; ^3^Faculty of Business and Management, Universiti Teknologi MARA, Melaka, Malaysia

**Keywords:** diaspora, Java, Malaysia, personal names, Javanese

## Abstract

The personal name of the Malaysian Javanese Diaspora (MJD) grows and develops along with the history of dynamic life, thought, social, and cultural developments. This research specifically aims to reveal and explain the process of anthroponymy evolution of the Javanese-Malaysia diaspora personal name system from historical, social, cultural, and linguistic characteristics aspects and the meaning of personal names that developed due to the cultural shifts and changes. This research utilizes data sources from lists of names of Javanese diaspora communities and efforts to extract names from MJD people who are in the locations of the states of Johor, Selangor, and Melaka in Peninsular Malaysia. Data were collected using content analysis techniques and in-depth interviews through discussions and Focus Group Discussions (FGD) as well as informal meetings directly at the research location. The results of the study show that the forms of names that appear in MJD society are strongly influenced by historical, social, and cultural phenomena that developed in the era before and after Malaysia’s independence. This phenomenon affects the characteristics of personal names so that it can be seen that there are phases of MJD people’s names, namely the initial arrival phase, the transition phase, the modern phase, and the latest phase. The results of this study can be used to find out the development of thought, spirit of life, and cultural traditions of a large MJD collective in Peninsular Malaysia.

## 1 Introduction

A personal name is a form of language that is understood and referred to by someone in the form of words, terms, or expressions that may be used to identify someone or something from another (Hofmann, 1993, 117). People’s names in various cultures around the world are often recognized as identities that distinguish a person from other people, animals, places, and other things that are known or talked about.

Javanese culture has the same traditional rules related to the matter of people’s names being always written in capital letters as a form of respect for those owning the name. Based on this understanding, suspecting that the name is related to issues outside the linguistic aspect is reasonable. Personal names in public life are not only concerned with the owner or his family (Bond and Forgas, 1984). A person’s name contains various other aspects, such as time, place, atmosphere or event, social status, history, and certain traditions. A name is a product of the society that can explain various things about the community itself. This is the starting point of this research, that personal names can refer to abstract ideas (culture, society, values, ideals, hopes, and prayers). Cavallaro (2004) states the following:

*These ideas do not simply refer to universal categories, but are closer to contextual meanings, based on symbols that are believed to be able to explain these ideas (p. 264)*.

Until now, research on Javanese personal names is still relatively rare compared to various linguistic, social, and cultural studies. Research on Javanese personal names, according to Uhlenbeck (2008), may be considered less interesting, narrow, and dry because there is not much that can be explored and revealed. The study of people’s names around the world always places the subject of personal names into a single paradigm from the perspective of linguistic structure (descriptive linguistics) without providing space and opportunities for other disciplinary perspectives to contribute their interesting ideas. As a result, name research falls into a narrow and dry field because it does not provide a choice of other points of view (Crystal, 1987).

The existence of a single paradigm in the research of people’s proper name systems can also produce very large errors, namely applying the meaning of names in a tautological way ‘repetition of declarative ideas, exaggeration, and lack of impact’. Moore (1954) stated that a name means the object and the object is the meaning. On the other hand, personal names are often confused with concepts, even though in the logic of language the two have different basic meanings (Charlesworth, 1959). This research is unique because it has a plural perspective to overcome the gap in knowledge about proper names which has been built, discussed, and understood by several language experts. This Proper name research has a strategic position in a multi-disciplinary context, namely language, social, historical, and cultural studies.

A historical source states that since the beginning of AD 13–14 the Javanese people have spread across Asia which is now known as Malaysia, Brunei Darussalam, Singapore, Thailand, and Vietnam (Allport, 1937). For example, the country of Malaysia until now has a fairly large population of Javanese descent, especially in the State of Johor. The Javanese, especially from the Kulon Progo and Ponorogo areas, migrated to the southern Johor region from the 18th century to the early 21st century. Most of the Javanese diaspora work in the plantation sector in Johor and Selangor and are popularly known as the Malaysian Javanese (Sariyan, 2015). This collective is people of pure Javanese descent who were born or emigrated to Malaysia. They form the majority of Malaysia’s population and Malaysian law considers them mostly Malay. Malaysia is home to the largest Javanese population outside Indonesia (Alatas, 2013).

Most Malaysians of Javanese descent have assimilated into the local Malay culture. They have used Malay as their mother tongue and first language (Shamsudin, 1969). This means that the Javanese language of their ancestors is their second language. The Javanese-Malaysian assimilation model falls under the ordinary category, namely through intermarriage with ethnic groups in the states of Johor, Selangor, Malacca, and other places. That is why they are then referred to as ethnic Javanese Malays under Malaysian law (Algeo, 2010). A similar situation exists with the Javanese in Singapore, where they are considered Malays. The interesting thing is that there have been various shifts and changes in the form of Javanese names to Javanese Malays, especially in the fourth and fifth generations. Of course, it becomes interesting to be able to know the social and cultural phenomena that occur, related to changes in orientation, world views, and cultural tastes that occur.

The preliminary research that has been carried out related to the subject of this study, among others: Suranto’s research (Suranto, 1983) entitled Study of Javanese Names, is a critical language study that provides a variety of preliminary information. Suharno’s research (1987) entitled Personal Names in Javanese Society, is a more complete study compared to previous research which summarizes various forms of tradition in conjunction with the procession of giving Javanese personal names, namely various traditional ceremonies (social spirit) performed to welcome the birth of a child. In addition, it is also mentioned in the research of Koentjaraningrat (1984), Ki Hudoyo Doyopuro (1997), Hadiwidjana (1968), Miftah (1998), and the Book of Primbon Betal Jemur Adam Makna (NN, 1934). These scientific works do not directly discuss the Javanese proper name system. However, it displays a long list of the best and selected Javanese names only from its analysis. In addition, Radjiman’s research (Radjiman, 1986) entitled History of Surakarta: An overview of political and social history specifically in Chapter IV discusses the tradition of giving names which is more oriented to the place-naming system (toponymy) in Surakarta.

Based on the statement above, Allport (1937) has the idea that a person’s name at the individual level is self-identity and there is also evidence that a person’s name has an influence on his life (Widodo, 2010). In this regard, the self-name of the Javanese-Malaysian diaspora community needs to be studied in depth so that the development of their life can be known and understood with certainty. This research is also expected to better see the phenomenon of mutual influence (borrowing process) between Javanese culture and Malaysian culture from the aspect of language, especially people’s names. In addition, it also has an important meaning for understanding the development of cultural tastes, traditions, and worldviews of the Javanese-Malaysia diaspora community. This has an impact on better knowledge of the growth of social, educational, artistic, and cultural models from time to time of the Javanese-Malaysian diaspora community from the perspective of using their names.

### 1.1 Theoretical basis

#### 1.1.1 Javanese in Malaysia

Javanese migration to Malaysia has occurred since the colonial era. Since the 1500s, many workers from Java were sent to the Malay Peninsula as manual labor in oil palm and rubber plantations (Miyazaki, 2000). Over time, the plantation companies in the Malay Peninsula continued to experience growth so they opened recruitment for Javanese people who wanted to work there. Therefore, the British opened a recruitment office in the big cities of Java to obtain workers from there (Liow, 2015). The Javanese population was indeed the main target for the colonial government to be used as laborers because in quantity the population was large and in terms of quality, they were able to do menial work. This migration continued until the 20th century. One of the areas in Java where many people were sent abroad was Klaten Regency, Central Java. In 1920, many residents in the Bayat District, Klaten, registered themselves as workers for European companies. The workers who pass the selection will then be sent to their migration places, one of which is Malaysia. Since the British colonial era in the Malay Peninsula, the capital city of the state of Selangor, Shah Alam, Malaysia, has been visited by many Javanese (Kamil and Noriah, 2011).

The Javanese workers lived in Malaysia for a long time, causing them to marry other fellow workers, thus forming a Javanese community, such as the one in the Batu Pahat area, Malaysia. According to the results of the 1950 Malaysian population census, most people born in Malaysia came from the island of Java, namely 189,450 people (Shamsudin, 1969).

#### 1.1.2 Characteristics of Javanese people’s names

Uhlenbeck (2008) in his research on the morphology of the Javanese language conveys in detail the systematic characteristics of the Javanese personal names. The step taken is to create a model for grouping Javanese names and the naming process. Uhlenbeck explicitly stated that the study of personal names is an interesting opportunity and becomes a subsystem that has clear boundaries in the overall linguistic structure. This research is a synchronous study for two reasons, namely: (1) The name data used is limited to the names of the Javanese people before 1950. The social background of the people is not captured (the color of social stratification), namely between the farming community, *priyayi*, and the royal family. (2) Javanese personal names are divided into six different groups based on gender (sex), social class, and the existence of a first and second name, especially for men’s names (Table 1).

**Table 1:** Classification of names according to Uhlenbeck.

FL	F
M1L	M1
M2L	M2

F (female) denotes women, M (male) denotes men, L (low) denotes groups of people who have a low social class, and practice numbers 1 and 2 to denote first and second names. The six sub-groups of names can be paired into 3 groups, namely: FL names with M1L, F names with M1, and M2L names with M2. FL and M1L.

#### 1.1.3 Meaning of Javanese names

Discussions about the meaning of names in the perspective of linguistics can be carried out from three focuses of attention, namely: (a) linguistic signs (in the form of phoneme series), (b) concepts (concepts belonging to phoneme series), and (c) referents (outside the linguistic field). It is also important to pay attention to symbols, which are any objects or events that refer to something. Crystal (1987) further conveyed that there are three important elements involved: the symbol itself, the existence of one or more references, and the relationship between the symbol and the reference. Furthermore, Spradley (1997) these three elements are referred to as Basic Symbolic Meanings. Ogden and Richard (1996) in The Meaning of Meaning state that meaning can be understood by looking at the relationship between the three components, namely designatum (concept, the essential component of lexical meaning), word (expression or form of the word), and with denotatum (referent). The relationship of the three components of meaning is shown in the diagram as follows.

Related to the theme of the name different possibilities occur. Leech (1983) provides a simple solution by knowing the thematic meaning, namely the meaning that appears as a combination of the meanings of its parts. The meaning of the name is communicated by the way the name giver organizes the message.

## 2 Scope and methodology

This research involved registering community names in three (out of nine) states of Malaysia, namely Johor, Selangor, and Malacca. In the three states of Malaysia, more than 20% of the population is a community of Javanese descent. However, the language used by people of Javanese descent in Malaysia is generally Malaysian. However, in an informal setting, Javanese is still used as a spoken language, especially by the adult and older generations. Most of the children and youth have used the Malaysian language.

**Figure 1:**
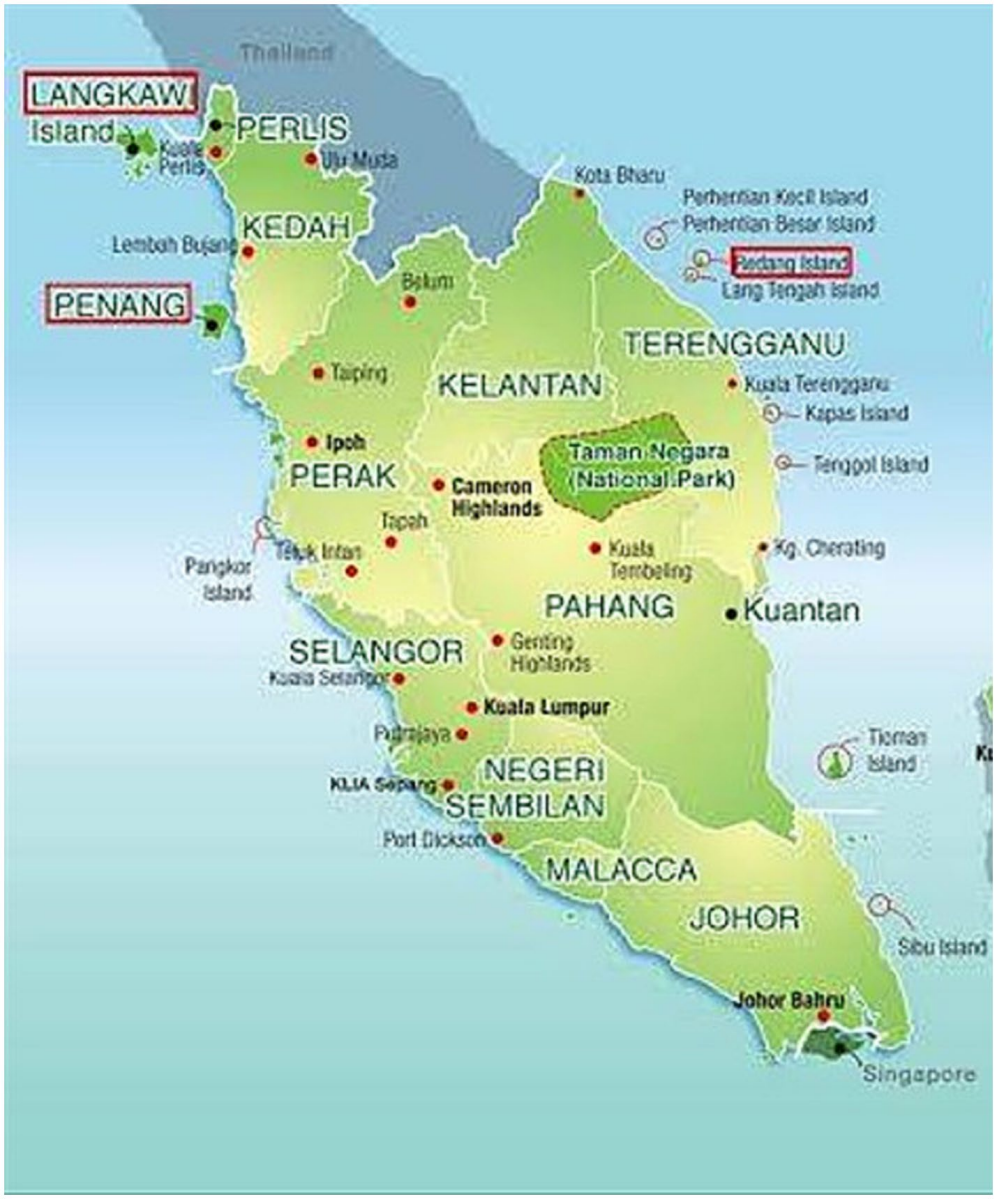
Peninsular Malaysia region.

**Figure 2:**
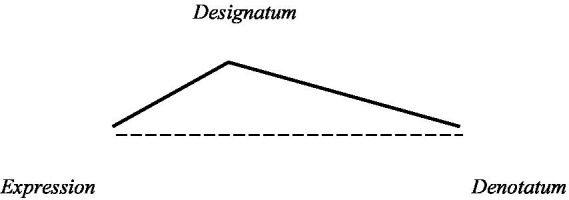
Triangle meaning according to Ogden and Richard (1996).

The location of each region is close to each other and are both located in the Peninsular Malaysia Region (Figures 1, 2).

Lists of personal names were obtained from schools at various levels (368 names) and from civil registration offices in the three research locations (604 names). The two sources are combined (972 names) so that the data classification can be arranged as follows (Table 2).

**Table 2:** Classification and distribution of personal name data in research locations.

Age	%	Total	Malaka	Johor	Selangor
3–10 years old	12	117	13	12	14
11–20 years old	15	147	22	16	11
21–35 years old	23	224	82	53	89
36–60 years old	32	311	144	136	79
>60 years old	18	174	72	58	44

The characteristic of the information contained from the register of personal names shows the unique linguistic construction of the Javanese diaspora in Malaysia, including: specific tribal patterns, the diversity of elements, the meaning of personal names, and the characteristics of up-to-date personal names. This is why we need registers of personal names that include the names of grandparents, fathers, and mothers to define the onomastic landscape over generations.

This name register continued to grow when interviews were conducted with several informants who were considered to know the research problems. Interviews were conducted individually and in groups (Focus Group Discussion) which were conducted 3 times, and each was attended by 6–8 people to find out the social, historical, and cultural background related to the development of the forms and meanings of personal-names used by diaspora communities. Among the 21 informants, 6 key informants have a social position in society. Interviews with key informants were conducted to dig deeper into the names’ motivations, expectations, and cultural tastes. The analysis in this research is divided into two, namely social analysis and linguistic analysis. The two are closely related because the birth of new forms of names cannot be separated from social, cultural, and social developments that move very dynamically in life (Table 3).

**Table 3:** Characteristics of key informants in research.

No	Names	Gender	Age	Social position	Place of residence
1	*Daim Darson*	Male	48	General public	Malaka
2	*Shafinar*	Female	55	Academics at UiTM	Malaka
3	*Johar Bin*	Male	62	Figures of the Malaysian Javanese Diaspora	Johor
4	*Afzan*	Male	47.	Malay community leaders	Johor
5	*Zaini*	Male	58	Academic, Professor of Politics and Social Affairs	Selangor
6	*Fuad*	Male	63	Academic, Professor of Politics and Social Affairs	Selangor

## 3 Discussion

### 3.1 Social dynamics of the development of the Malaysian Javanese diaspora names

The dynamics in the MJD context are related to two issues, first, the process of social interaction of the Javanese community with the local population in the research locations of Peninsular Malaysia (Malacca, Johor, and Selangor) which turned out to be very hetero-social, consisting of indigenous Malays and migrants from Bugis-Sulawesi, Palembang, Aceh, Padang, and small communities from Madura. Of course, the number of collectives outside Malay is limited. The process of social interaction greatly influenced the emergence of changes in the form of Javanese names to Malay Islamic names. Second, is social interdependence, namely the process of mutual synergy and mutual influence between group members. The process of interaction and interdependence leads to adjustment efforts, mutual influence, and understanding in conditions that are constantly moving, developing, and adapting to circumstances.

Starting from these conditions, this research needs to consider several things related to the social phenomena behind the arrival of Javanese people in Malaysia, social strategies that emerged in the adaptation process, and modern-global influences (name trends) to date. All three are seen as able to provide an adequate explanation regarding the development of MJD’s self-name to date.

### 3.2 The phenomenon of the arrival of the Javanese in Malaysia

Waves of arrival of people from various regions to the Peninsular region have occurred centuries ago. This group of migrants comes from Javanese, Acehnese, Bugis Makassar, Palembang, Padang, Madura, and other groups from Riau, Nusa Tenggara, etc. Various ethnic groups who came to the Peninsula had different aims and objectives. From the data collected, the Javanese people came to the peninsula for various reasons, namely:

1. They trade in various forms of trade, they mingle and interact with the indigenous people, live and eventually settle down and receive recognition from the authorities (the British Empire and the local government) along with other trading groups who come from Padang, Palembang, and Madura.
2. Migrating, looking for a better life. They come and get placement permits by clearing wilderness forests and farming until they form *mukim* communities that are legal and recognized with other migrant communities. They inhabit a single area with ever-growing boundaries of ditches or rivers.
3. The spread of Islam, this community came from religious groups (Islamic boarding schools).
4. Fleeing from the pursuit and threats of the Dutch colonial government in their homeland in Java.

Until 1940, the Javanese people in the Peninsular region (Malacca, Johor, and Selangor) still used their original Javanese names. They live in groups in new areas bounded by ‘small river’ ditches or rivers, including Sarang Buaya River, Ponoroio Trench, Bagelen Trench, Kemang Trench, Resik Trench (Gresik) which also form the boundaries of the area.

*This is understandable because since ancient times the Javanese have always been characterized by the existence of “water” and “surau or mosque.” They are people who are diligent in clearing new forests and lands, are open to the association, and can be role models for migrants from other areas, such as the Bugis, Mandailing, Sumatra,* etc.*”* (Johar, Muar-Johor, 16 July 2023).

This area continues to grow to form a settlement. In settlements, it is led by a *lurah* ‘village head, Javanese term’ or *Pengulu*, and a *Naib* who has the task of taking care of marriages. The term *Lurah* until the end of the 19th century can still be found. However, since the issuance of the 1913 British Royal Regulation concerning Land Ownership by *Bumiputra* and Malays, the term has changed to *Penghulu* who controls one *mukim* area with several villages and is responsible to the kingdom. The pattern of social grouping at this time made it easy to find sites of old Javanese names (Table 4).

**Table 4:** Original Javanese names in Malaysia 1930–1965.

No	Real name form	Arrival year	Gender	Origin name
1	*Pawiro(sonto)*	1930s	M	Ponorogo, Jawa Tengah
2	*Paimin*	1940s	M	Banyumas, Jawa Tengah
3	*Juwariyah*	1930s	F	Ajibarang, Jawa Tengah
4	*Wagini*	1950s	F	Sumberagung, Wonogiti
5	*Sapon*	1930s	M	Pijiharjo, Wonogiri
6	*Paitinah/Fatimah*	1930s	F	(?), Sragen
7	*Tohar*	1960s	M	Dimong, Purworejo
8	*Mingan*	1950s	M	Siman, Ponorogo

Source: Interview, 5 July 2023, Muar Johor.

### 3.3 MJD’s name: mirror of Javanese adaptive behavior in Malaysia

The change of Javanese names into Islamic Malay names occurred evolutionarily in line with social, political, and historical developments, position, and citizenship processes. The long process of adaptation and mutual acceptance between the local people and the immigrant collectives took place well and “melted” in the form of personal names.

*“In the past, our grandparents (Javanese) came for the first time, so it was not easy. They sailed across the Ocean, opening up plantations and the wilderness of the Johor Region. We live with residents of Malay origin and fellow immigrants from Sulawesi and Banjar. We try to understand and accept each other. Learning the language of the Malay population, adjusting to the new culture, and giving children Malay names. That’s how we adapt”* (Shaifuddin Bin Sapon, interview, 9 July 2023).

The results of the interview were developed by examining existing documents so that the factors influencing the name change could be identified, including government factors (British and Royal Malaysian Government) and certain social movements. Several social phenomena that have been recorded indicate the presence of four other social factors, namely:

1. The issuance of the Law on Malayu Land and Land Ownership for *Bumiputra* in 1913. This official regulation further accelerated the transfer of the status of the Javanese population to become Javanese Malay.
2. After World War II, in 1948 the British Government conducted a population re-registration because of Communist activity in the Peninsula which was about to revolt. The British Empire distinguished the population on the Peninsula into three major groups, namely Malays, Chinese, and Indians. Javanese descendants are included in the Malay group along with other collectives such as Sulawesi, Banjar, Aceh, Padang, etc. This phenomenon became a strong impetus for the name change.
3. The influence of the Islamization movement in the Peninsula from the late 1960s to the 1980s. Javanese names were changed by civil registration officers to facilitate the process of social adjustment, land ownership, and residence status of the Malays. Most strong shows occur in schools.

Change of Javanese name after returning from performing Hajj in Arabic. Javanese names such as *Sugimin, Tarmo, Tumirin, Ginem,* or *Rubiyem* were changed to new Islamic names, namely *Abdol, Ibrahim, Mohamad Zain, Fatimah,* and *Zubaidah.*

### 3.4 Linguistic construction of the names of the Malaysian Javanese diaspora

The name of the Malaysian Javanese Diaspora (MJD) is a speech that has an interesting form, structure, and meaning. Based on the data collected, it appears that there are prominent characteristics in terms of writing names, including (1) Linguistic Construction of MJD, (2) Patterns of Ethnicity of MJD’s name, (3) Diversity of elements of MJD’s name, and (4) Meaning of MJD’s name. The four characteristics are connected, although each can be distinguished in terms of scope of study, but they are interrelated in the form, meaning, and meaning that are unique to MJD’s name.

### 3.5 Linguistic construction MJD

During the research on MJD’s name data, many forms of the names *Nur* ‘light’, *Siti* ‘female/mother’, and *Zahra* ‘bloom, the flower of the world’ was found, which are popular as elements of female names. Elements of the names *Muhammad*, *Abdul*, and *Afiq* are popular as elements of male names. Elements of this name are found as the names of children to adults who have the basic construction of a singular noun. The characteristics of MJD’s name which are basic or single construction can then be called monomorphic construction names. In other forms, there are also variants of single element forms which have been combined with other basic morphemes to form new element names such as *Nurliyana*, *Fikrirullah*, *Jannatunnaim*, and *Nurhaikal*. This series can be called a polymorphemic construction name because it has more than one morpheme element.

The polymorphemic construction in the case of MJD’s name is unique because the double morphemes are both intact and independent. Two provisions apply to the merging of the two basic elements, namely joining as a whole and experiencing syllable dissolution events so that they become one element of a new name. The name *Nurhaikal* is an example of a complete merger between *Nur* ‘light’ and *Haikal* ‘to grow tall’. Likewise, the name *Jannatunnaim* is a combination of *Jannatun* + *Naim* ‘the most beautiful heaven’. Unlike the name *Fikrullah* which is formed from two basic elements, *Fikri* is ‘smart, clever’ and Allah ‘Lord Allah’.

The emergence of monomorphemic and polymorphemic patterned name forms indicates that there has been a change and development in the form of the MJD name from the construction of the original Javanese name which had occurred over the previous seven decades. In the era of the 1950/1960s, the names of Malaysians of Javanese descent still showed a typical Javanese construction, namely a combination of independent morphemes and independent morphemes (Su-) as shown in Table 5.

**Table 5:** Polymorphemic construction of MJD name old age group.

Name (Age)	Morphemic process	Name meaning
*Sukiman Bin Sarmani* (72 years)	*Su + kiman* ‘good, deed’	Men who have good deeds and attitudes
*Sukarti Binti M Ridwan* (78 years)	*Su + karti* ‘good appearance’	Woman with a good face & Appearance
*Supardi Bin Bai* (71 years)	*Su + pardi* ‘good lineage’	A man of good ancestry.
*Suwagiyo Bin Paimin* (69 years)	*Su + Wagiyo* ‘a good friend’	Men as good (life) friends.

**Figure 3:**
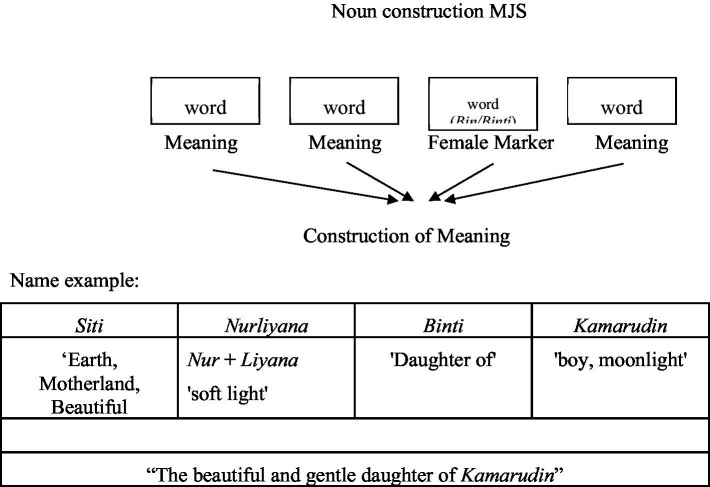
MJD name construction.

Based on data in the field, people in Malacca, Johor, and Selangor of Javanese descent who are more than 65 years old rarely bear phraseologically constructed names (construction of groups of words) as they often do in Java. The Javanese female name *Mustikaningrum Idris* has a complex phraseological construction, namely: *Mustika* ‘jewel’ + (n)*ing* ‘to’ + rum ‘fragrant, heart’; *Idris* (shows the daughter of *Idris*). This construction is rarely found in the name MJD. Most of MJD’s names aged 5–50 years are constructed as words, not groups of words, one name consists of several elements of the whole name (Figure 3).

The construction of MJD’s name is unique because the various forms of the name’s elements combine to form a beautiful monomorphemic series (Uhlenbeck, 2008; Shanmuganathan, 2021). This is very much felt at the time of interpreting the meaning of the name. In addition, different and joined morphemes always have certain meaning characteristics based on their phonemic forms which are permanently related to their meaning characteristics (Uhlenbeck, 2008). Of course, it is important to note that the name MJD in terms of form (phonetics) and meaning (semantics) has developed from the old form (Javanese) to a form that is typically Malay (Malaysia) through ways that are also classified as typical.

### 3.6 MJD’s syllable pattern

The syllable pattern of MJD’s name is an important part of the linguistic analysis system, including (1) knowing the length and shortness of the name, (2) knowing the relationship pattern between parts, (3) knowing the linguistic beauty of MJD’s name and (4) as a way of interpreting a name by paying attention to the unit of form in speech (Suharno, 1987; Verhaar, 1998). From the collected data it is clear that MJD’s name is quite varied, in the construction of elements, short names only consist of one to three syllables. However, some names show a tribal pattern that consists of four to six syllables.

Table 6 shows that MJD names mostly consist of one to four syllables. The tendency is that the more the number of syllables in the name, the less its usage is found in society. In addition, most of the four-syllable MJD names tend to be found in modern names (after the 2000s). These two findings show a slightly different direction from the development of Javanese names in Indonesia. Modern names in Java tend to be long there are many names consisting of five and six syllables. Examples of five-syllable names include *Kurniawanto*, *Sugiartoyo*, *Sulistiono*, *Wahyidiono*, and *Damaringalam* (male names) and *Elminangkani*, *Kurniawati*, *Anggitasari*, *Susilowati*, *Mardihutami*, *Ambarawati* (female names). Javanese male names have six syllables, for example, *Taufikurahman* and *Kusumawardhana*, while *Nilawatiningsih* and *Kumaralalita* are examples of female names.

**Table 6:** Number of syllables in the MJD name.

Syllables	Male name	Female name
One	*Abu, Kas, Mak,*	*Nur, Noor, Mas,*
Two	*Omar, Zaky, Asyraf, Mirza, Hakim, Adam, Cheman, Azlan, Azrin, Adeb*	*Salmah, Habsah, Siti, Balqis, Yuhra, Uzma, Normah*
Three	*Hairi, Iskandar, Hakimi, Muhammad, Mikhail, Aiman*	*Aqilah, Nafisah, Ezuma, Hamidah, Hidayati,*
Four	*Hafizuddin, Zahrulnizam, Norashidi, Hishamuddin*	*Qaisahrah, Qairina, Umairah, Nurfareena*

### 3.7 The diversity of elements of MJD’s name

Analysis of the diversity of elements of the MJD name system has an important position to research because it forms the basis for understanding the position of the MJD name as a label, sign, and identity of a person or group that differentiates them from other people (Hofmann, 1993) and certain groups of people (Lehrer, 1999). Uhlenbeck stated that a name can only be used to refer to someone, even though there is the same name in society (Akinnaso, 1980). The male names in the Johor and Malacca areas are quite large using the Muhammad element which is oriented singly to the Prophet Muhammad. This is easy to understand because Malaysia is an Islamic country, and the majority of the population is Muslim. However, it is interesting that the writing of elements of the name *Muhammad* has several acronym variants, namely: *Muhammad* (the form is still intact), *Muhamad* (written in “m” one), *Moch*., *Mohd*, and *Mohamad*. The same thing happened to the element of the name *Nur* ‘light’ appearing with several variants, namely *Nor* and *Noor*. Interestingly, elements of the name *Nor* often appear as components of the elements of the names *Noriah*, *Normah*, *Norwadiah*, *Norakmar*, and *Noraini*. This also often occurs in name series in Java, Asia, and Europe because personal names are social products (Aleksieieva, 2021).

The diversity of the elements of MJD’s name is inseparable from the corridors of norms, traditional conventions, bonds of national spirit, adaptability, the spirit that is built, and the cultural tastes of the people. Sutarjo (2007) states that the diversity of personal names is a way for the Javanese diaspora to engage in dialog and adapt to the local community, natural and social environment through language, behavior, and ideas (see Langendonck, 1985). According to Lieberson (1982), the driving factors for the birth of various names are influenced by: (1) the level of parental knowledge of names, (2) the level of social status, and (3) institutional, religious, and world norms. It seems that the opinions of Langendonck (1985) and Lieberson (1982) especially concerning social status issues have less and less influence on personal names. The main causative factor is the changing times and advances in science and technology which have given birth to new ways of thinking. The Javanese-Malaysian diaspora community in the last three decades has shown an increasingly better social position and role in Malaysia. They have blended in and become part of Malay society in form, spirit, meaning, and function which can be seen from the values, ideals, and expressive symbols of the names they bear (Kuntowijoyo, 2004; Thomas and Samjose, 2022).

The classification of the elements of MJD’s name was compiled based on data on the names of the Malaysian Javanese diaspora community in Johor, Malacca, and Selangor by grouping them based on similarities in form, nature, and meaning which show the same syntactic behavior and relationship characteristics (compare Lawson, 1984). Based on the data collected, the elements of MJD’s name can be classified into seven categories as follows (Table 7).

**Table 7:** Classification of the seven categories of MJD names.

Source name	Original form	Change form
*Asma’ul-Husna*	*Arrahman* *Arrahim*	*Rahman* *Rahim, Rakhim, Rakim*
Names of the Prophets	*Adam, Idris, Ibrahim, Ismail, Yusuf, Ayyub*	*Adam, Idris, Ibrahim, Ismail, Yusuf, Yusoff, Yusop, Ayub*
Companions of the Prophet Muhammad	*Abu Bakar, Umar bin Khathab, Utsman bin Affan, Ali bin Abu Thalib*	*Abu Bakar, Oemar, Umar, Usman, Oestman, Ali, Tolib, Talib*
Ten people guaranteed to enter heaven Allah SWT	*Sa’d bin Zaid bin Naufal, Abu Ubaidah Amir bin Al-Jarrah, Sa’d bin Abi Waqqash, Abdurrahman bin Auf, Zubair bin Awwam, Thalhah bin Ubaidillah*	*Zaidan, Ubaidah, Ameera, Ameer, Amir, Zubaer, Zuber, Tolkah, Thalkah,*
The Prophet’s Wives	*Khadijah, Saudah, Aisyah, Hafshah, Zainab, Ummu Salamah, Juwairiyah, Shafiyah*	*Kodijah, Khodijah, Qotijah, Hafsah, Hafizah, Zalamah, Juwariyyah,*
Prophet’s sons and daughters	*Al-Qasim, Abdullah, Ibrahim, Zainab, Uqayyah, Ummu Kultsum*, and *Fathimah*	*Khosim, Abdullah, Zainab, Ukayyah, Kulsom, Fatimah*
Association of virtue, nobility, chastity,	*Asyam* ‘a noble leader’, *As’ad* ‘a happy person’, *Anwar* ‘a good person’, Amin ‘trustworthy’	*Aszam, Azam, Asad, Anwar, Ameen, Amin, Ammin, Shaifuddin,*
Another form	*Ariff, Almeer, Arsyilla, Aidyn, Bahran*	*Arif, Areef, Almeer, Almira, Arsilla, Arsyilla, Bahran, Aidin*

### 3.8 The meaning of MJD names

The data shows that MJD’s name is related to the knowledge, truth, meaning, and reason of the giver and bearer of the name. That is, there is a relationship between the name as a statement with the wishes, hopes, meanings, and intentions as the circumstances described (Cavallaro, 2004). The next name is considered as a way or method that fulfills the truth-conditions (Langendonck, 1985). This is the basis for understanding the name MJD, which contains abstract ideas that melt away, reflections of the situation and conditions of the social and cultural environment.

MJD’s name has an interesting meaning. Related to this, Ullman (1977) states that *“..... the person’s name identifies and does not signify ‘understand’*. This is the basis used to reveal the deepest meaning of a name. The frame of reference must be determined based on the language and culture of the owner of the name so that the contents of the code and conventions can be known with certainty (Gardiner, 1954; Ullman, 1977; Crystal, 1987). The terms of reference are (1) identification, (2) uniqueness, (3) denotation, and connotation, (4) distinctive sounds, and (5) grammatical criteria. The five terms of reference can be explained as follows.

First, the name MJD is a marker of the individual and collective identity of the community. The name is given to someone not occasionally to give a mark for him, but as a label idea that distinguishes him from other people (Xu, 2020) so that the name always refers to the person he is in. Second, in the MJD name, we find many elements of the names *Muhammad, Nur, Noor, Siti, Adam, Qaseh,* etc. But still, one name is only for one person, so even though the form of the name is the same. Third, a name is understood from its denotative meaning and then its connotative meaning. The element of the name *Akbar* in the name *Akbar Bin Muhamad Huzni* means ‘big’, describes a big situation, and connotatively always big all the time. The same goes for the name *Nasrah Binti Mahmud*, of course, it means that those who have the name will have peace in their lives, be able to become educators for the younger generation, be able to set an example, etc. So, it turns out that the meaning of the name does not refer to what is designated, but to what is connoted.

Fourth, the proper name is an identification achieved by its distinctive sound. The basic meaning of a name may be denied by the giver because of other purposes which are classified as special, unique, and an exception which is then called the ‘distinctive’ meaning of the name which is the ‘other face’ of a form of speech called self-name. Fifth, grammatical specificity is one of the grammatical criteria of personal names. The unification between components and elements even though it is not grammatical, it is still a person’s name.

Based on the results of the analysis of the linguistic aspect, namely the classification of the elements of the name, the diversity of elements, and other relevant tendencies, it can be seen that the basis for selecting the elements of the name MJD, namely (1) religion and beliefs, (2) social and cultural considerations, (3) considerations of lineage, origin, family, or historical setting of the sufferer, and (4) accommodates all the hopes, wishes, and prayers of parents. The four basic elements of the name selection make it easier for us to understand why elements of names such as *Muhammad, Nur, Noor, Siti, Adam,* and *Qaseh* often appear. The element of the name *Muhammad* (with the variants *Muhamad, Mohd,* and *Mohamad*) is recognized as an element of the popular name and is, therefore, the most common male name in Malaysia. The element of the name *Nur* (with the variants *Noor* and *Nor*) can be recognized as the element of the most popular female name in Malaysia.

## 4 Conclusion

The results of the research analysis clearly show that there is a strong relationship between the linguistic form of a proper name and various social, and historical backgrounds and various cultural phenomena of the MJD community. The results of the search and critical analysis show that the personal name MJD has clear characteristics, including (1) having a clear gender marker by adding *bin* names for men’s names and *binti* for women’s names behind the person’s name. (2) Mention part or all of the parent’s name after son or daughter. (3) MJD’s personal name shows a strong Islamic image (8 categories), of course, there is a series of names that show general meanings (*Johar* ‘name of the tree’, *Kemis* ‘name of the day’, *Slamet* ‘safe’). (4) The use of the letter Z as an esthetic variation of the name is very prominent compared to the names in Java (*Zahid-Sahid*, *Syazuwin-Aswin*, *Syazwana-Sas(r)wana*, and *Izdihar-Ishar*).

The second conclusion is that the development of the form, function, and meaning of a proper name is influenced by current trends. MJD’s personal-name grows and develops in line with the history of Malaysia. There are at least four phases of the development of names that can be conveyed, namely (1) the initial phase of the arrival of the Javanese before the independence of Malaysia (1957), (2) the period of adaptation and transition of name forms, (3) the period of development of modern forms, and (4) the period of modern names. The MJD name phase can be described as follows.

1 Initial arrival phase

Until the 1940s MJD’s personal name still used the original and intact form of the Javanese name (see Table 4). The inclusive attitude of immigrant communities from Java Island facilitated the process of acceptance by indigenous people in Malacca, Johor, and Selangor. The use of this native name also shows clear cultural boundaries between natives and immigrants. The people of Javanese origin in the early days occupied the permitted areas and had ditches or river boundaries.

2 Transition and adaptation phase

This transition phase is conveyed from written data, namely the list of names of documents that have been collected. The form of transitional names is marked by the presence of two forms of names, namely Malay and Javanese names in one person’s name which are separated by the sexes *bin* (male name) and *binti* (female name). Malay names are used to refer to children’s names, while names after bin/binti are the names of parents who still have Javanese names. This name series existed until the 1990s. A series of transitional names can be called mixed names, for example: *Jais Bin Gimin, Azlam Bin Ngasman, Saifuddin Bin Sapon, Suhaella Binti Miskam, Juliyyana Binti Aliman*.

3 Modern phase

The modern phase is marked by the presence of names that match current trends. The elements of MJD’s names at this time were also influenced by the trend of names outside the country. For example, the names *Adeb, Danish, Mirza, Fares,* and *Uzma* are a category of modern names that are mostly used by children aged 11 to 18 years.

4 Advanced phase

The Advanced Phase is used by children aged 1–10 years in the MJD community. This phase is marked by updated modern names. The forms of the names *Adeb, Danish, Mirza, Fares,* and *Uzma* above actually have other variant forms, namely *Adeeb, Dannish, Mirzza/Mirzha, Farres,* and *Uzmma*. The difference is the age of the name holder. Advanced names are often used by children aged less than 10 years.

## Data availability statement

The original contributions presented in the study are included in the article/supplementary material, further inquiries can be directed to the corresponding author.

## Author contributions

SW: Conceptualization, Data curation, Formal analysis, Funding acquisition, Methodology, Writing – original draft. SS: Investigation, Supervision, Writing – review & editing. BM: Resources, Supervision, Writing – review & editing. SI: Investigation, Resources, Validation, Writing – review & editing.

## References

[ref1] Akinnaso N. (1980). The sociolinguistic basis of Yoruba personal names. Anthropological Ling. 22, 275–304.

[ref3] Alatas S. H. (2013). The myth of the lazy native: a study of the image of the Malays, Filipinos and Javanese from the 16th to the 20th century and its function in the ideology of colonial capitalism. UK: Routledge.

[ref4] Aleksieieva N. (2021). Associative identification of personal names: a cognitive approach. Wisdom 2:18. doi: 10.24234/wisdom.v18i2.507

[ref5] Algeo J. (2010). Is a theory of names possible? Names 58, 90–96. doi: 10.1179/002777310X12682237915106, PMID: 37963266

[ref6] Allport G. W. (1937). Personality: A psychological interpretation. New York: Holt.

[ref7] Bond M. H. Forgas J. P. (1984). Linking person perception to behavioral intention across cultures: the role of cultural collectivism. J. Cross-Cult. Psychol. 15, 337–352. doi: 10.1177/0022002184015003006

[ref8] Cavallaro D. (2004). Teori kritis dan teori budaya. Yogyakarta: Niagara.

[ref9] Charlesworth M. J. (1959). Philosophy and linguistic analysis. Pittsburgh: Duquesne University.

[ref10] Crystal D. (1987). The Cambridge encyclopedia of language. London: Cambridge University Press.

[ref100] Gardiner. (1954). The theory of proper names. London - New York - Toronto: Sage.

[ref11] Hadiwidjana. (1968). Nama-nama Indonesia. Yogyakarta: UP Spring.

[ref12] Hofmann T. R. (1993). Realms of meaning. New York: Longman Publishing.

[ref14] Kamil K. Noriah K.. (2011). Masyarakat Keturunan Jawa Johor. Johor Bahru: Yayasan Warisan Johor.

[ref15] Ki Hudoyo Doyopuro (1997). Pakubuwono Ke IV Cipto Waskito Ngelmu Mistik Terapan. Semarang City: Dahara Prize..

[ref16] Koentjaraningrat. (1984). Kebudayaan Jawa. Jakarta: Balai Pustaka.

[ref17] Kuntowijoyo. (2004) Raja, priyayi, dan kawula: Surakarta 1900–1915. Yogyakarta: Ombak.

[ref18] Langendonck W. V. (1985). Pragmatics and iconicity as factors explaining the paradox of quantified personal names. Names 33, 119–126.

[ref19] Lawson E. D. (1984). Personal names: 100 years of social science contribution. Names 32, 45–73. doi: 10.1179/nam.1984.32.1.45, PMID: 37483944

[ref20] Leech G. N. (1983). Semantic. New York: Pinguin Book Ltd.

[ref21] Lehrer A. (1999). Proper name: linguistic aspect. Oxford: Elsevier.

[ref22] Lieberson S. (1982). A matter of taste: How names, fashions, and culture change. George Washington University, Gale Group: US.

[ref23] Liow J. C. (2015). "The politics of Indonesia–Malaysia relations – kinship and indo-Malay historiography (kinship and the pre-colonial regional system)". England: Taylor & Francis.

[ref24] Miftah F. (1998). Sekelumit nama-nama moslem. Yogyakarta: Andhara.

[ref25] Miyazaki K. (2000). Javanese Malay: between adaptation and alienation. J. Soc. Southeast Asia. 15:2000.

[ref26] Moore G. E. (1954). Principle ethics. Cambridge: The University Press.

[ref27] NN. (1934). Kitab Primbon Betal Jemur Adam Makna.

[ref28] Ogden Richard. (1996). The meaning of meaning. London. Routledge & Kegan Paul Ltd.

[ref29] Radjiman. (1986). Sejarah Surakarta tinjauan sejarah politik dan sosial. Surakarta: Krida Aksara.

[ref30] Sariyan A. (2015). “Persepsi Keturunan Jawa di Malaysia Terhadap Bangsa Jawa di Tanah Induknya dalam Konteks Keserumpunan Tamadun” in Makalah Seminar dan konferensi. Ed. Universiti Pendidikan Sultan Idris. Malaysia: Institut Peradaban Melayu, Universiti Pendidikan.

[ref31] Shamsudin H. Sistem Kekeluargaan di Kalangan Orang-Orang Keturunan. (1969). Jawa di Muar (Undergraduate thesis, University of Malaya).

[ref32] Shanmuganathan T. (2021). Names and naming practices of the Telugu in Malaysia. J. Onomastics 69, 34–42. doi: 10.5195/names.2021.2277

[ref33] Spradley P. J. (1997). Metode etnografi. Yogyakarta: Tiara Wacana.

[ref34] Suharno. (1987). Sistem nama diri dalam masyarakat Jawa. Yogyakarta: Depdikbud Daerah Istimewa Yogyakarta.

[ref35] Suranto A. (1983). Studi tentang sistem nama-nama Jawa. Surakarta: Fakultas Sastra UNS.

[ref36] Sutarjo. (2007). Kajian Budaya Jawa. Surakarta: Jurusan Sastra Daerah UNS.

[ref37] Thomas C. N. Samjose B. (2022). ““My name is …”: Picturebooks exploring linguistically and culturally diverse names” in Names: A journal of onomastics, vol. 70, 19–30. doi: 10.5195/names.2022.2468

[ref38] Uhlenbeck E. M. (2008). Kajian morfologi bahasa Jawa. Jakarta: Djambatan.

[ref39] Ullman S. (1977). Semantics, an introduction to the science of meaning. Basil Blachwell: Oxford.

[ref40] Verhaar J. W. M. (1998). Teori Linguistik dan Bahasa Indonesia. Yogyakarta: Kanisius.

[ref41] Widodo S. T. (2010). Nama Orang Jawa: Kepelbagaian Unsur dan Maknanya. Int. J. Malay World and Civilisation 28, 259–277.

[ref42] Xu X. (2020). Exploring the logic of name changes and identity construction: a reflective self-narration of assimilation expectations. Names 68, 32–41. doi: 10.1080/00277738.2018.1452937

